# Ambroxol confers neuroprotection against scopolamine-induced Alzheimer’s-like pathology by modulating oxidative stress, neuroinflammation, and cognitive deficits via Nrf-2/JNK/GSK-3β signaling pathways

**DOI:** 10.3389/fnagi.2025.1607289

**Published:** 2025-07-23

**Authors:** Waqas Ahmad, Kyonghwan Choe, Riaz Ahmad, Tae Ju Park, Myeong Ok Kim

**Affiliations:** ^1^Division of Life Science and Applied Life Science (BK 21 Four), College of Natural Sciences, Gyeongsang National University, Jinju, South Korea; ^2^Department of Psychiatry and Neuropsychology, School for Mental Health and Neuroscience (MHeNs), Maastricht University, Maastricht, Netherlands; ^3^Department of Cell Biology, Albert Einstein College of Medicine, Bronx, NY, United States; ^4^Alz-Dementia Korea Co., Jinju, South Korea

**Keywords:** Alzheimer’s disease, scopolamine, ambroxol, oxidative stress, neuroinflammation, synaptic dysfunction.

## Abstract

Alzheimer’s disease (AD) is the most common and costly chronic progressive neurodegenerative disorder, with the highest impact on public health worldwide. Pathological hallmarks of AD include progressive cognitive decline and memory impairment, dominantly mediated by oxidative neurodegeneration. Oxidative stress is commonly recognized as a key factor in the pathophysiological progression of AD. Despite significant advancements, a definitive and effective therapeutic intervention for AD remains elusive. In this study, we investigate the neuroprotective potential of ambroxol (Amb), known for its potent anti-inflammatory and antioxidant properties. Given ambroxol’s potential neuroprotective effects, we explore the underlying molecular mechanisms, explicitly examining its role in attenuating scopolamine-induced oxidative stress-mediated activation of the c-Jun N-terminal kinase (JNK) pathway, as well as its modulation of Akt and glycogen synthase kinase-3 beta (GSK-3β) signaling, which is a key contributor to neuroinflammation, synaptic dysfunction and neurodegeneration. AD pathology is induced by scopolamine administration, leading to excessive lipid peroxidation (LPO) and reactive oxygen species (ROS) generation, which leads to a decline in critical antioxidant proteins, including nuclear factor erythroid 2-related factor 2 (Nrf-2) and heme oxygenase-1 (HO-1). However, ambroxol treatment effectively attenuated oxidative stress by reducing the production of reactive oxidative species while restoring the expression of key antioxidant proteins. Similarly, ambroxol attenuated oxidative stress-induced JNK activation and modulated Akt and GSK-3β alterations. Immunofluorescence and western blot analyses revealed that ambroxol attenuated reactive gliosis by suppressing the expression of GFAP and Iba-1, alongside the downregulation of key pro-inflammatory mediators, such as IL-1β, TNF-α, and phosphorylated NF-κB (p-p65). Scopolamine also compromised synaptic integrity and induced deficits in memory formation and spatial learning. In contrast, ambroxol promoted synaptic integrity by upregulating the expression of SNAP-23 and PSD-95, thereby ameliorating scopolamine-induced impairments in spatial learning and memory.

## 1 Introduction

Progressive cognitive decline and memory impairment are defining characteristics of Alzheimer’s disease (AD), one of the most prevalent and complex neurodegenerative disorder. AD is a leading cause of disability and morbidity worldwide, significantly affecting the aging population, as advanced age is the primary risk factor for its onset and progression (Liu et al., 2017; Stephenson et al., 2018; Volkman and Offen, 2017). Pathological features of AD include degeneration of the brain’s cholinergic system, the formation of neurofibrillary tangles, and the accumulation of senile plaques (Zhang et al., 2023; Muhammad et al., 2019). The pathology of AD also involves neuronal loss, neuroinflammation, and oxidative stress, which contribute to progressive memory impairment and cognitive decline (Lee et al., 2012). Although each of these factors appears independent, they are intricately interconnected through the common oxidative stress pathway (Bai et al., 2022). Oxidative stress is a pivotal cellular response involved in AD and aging, resulting in excessive production of lipid peroxidation (LPO) products and reactive oxygen species (ROS). This imbalance surpasses the capacity of the antioxidant defense system, leading to cellular damage and accelerating neurodegeneration (Thingore et al., 2021). Oxidative stress plays a central role in multiple pathogenic pathways of AD, driving neuroinflammation, reducing the synthesis of antioxidant proteins, and contributing to synaptic dysfunction (Bai et al., 2022). Dysfunction of the cholinergic system results in a reduction of acetylcholine (ACh) levels, a key factor in the pathophysiology of dementia. Current therapeutic strategies focus on enhancing cholinergic neurotransmission by either increasing acetylcholine (ACh) levels or inhibiting its degradation through acetylcholinesterase (AChE) inhibition. Approved anti-alzheimer’s disease drugs, including acetylcholinesterase (AChE) inhibitors like tacrine, donepezil, and N-methyl-D-aspartate (NMDA) receptor antagonists, can alleviate symptoms, particularly in the early to moderate stages of the disease. However, their prolonged use may be linked to adverse effects (Hampel et al., 2018; Lahiri et al., 2004).

Scopolamine (Scop) is a well-known anticholinergic agent that acts as a non-selective muscarinic (M) receptor antagonist that efficiently crosses the blood-brain barrier. It competitively inhibits acetylcholine (ACh) binding to muscarinic receptors, resulting in learning and memory deficits, primarily by disrupting central cholinergic signaling (Muhammad et al., 2019; Drummond and Wisniewski, 2017; Rahimzadegan and Soodi, 2018; Zhao et al., 2024). Intraperitoneal injection of scopolamine induces cholinergic neuron degeneration and a reduction in acetylcholine (ACh) levels, primarily due to its antimuscarinic effect. This effect leads to diminished acetylcholine (ACh) activity and impaired cholinergic neurotransmission, which subsequently promotes the generation of reactive oxygen species (ROS) and lipid peroxidation (LPO). This further exacerbates oxidative stress, neuronal damage, neuroinflammation, and memory impairment, which are crucial factors in the progression of AD-like dementia (Wang et al., 2022; Abu Almaaty et al., 2021; Lu et al., 2018). Scopolamine administration induces behavioral and molecular features resembling those of Alzheimer’s disease and other neurocognitive disorders; therefore, scopolamine-treated animal models are widely used in neurocognitive research (Cheon et al., 2021). Numerous studies have demonstrated that scopolamine induces oxidative stress in the brains of rats and mice, leading to neuronal damage (Ju et al., 2021). Consequently, scopolamine-induced mouse models can partially replicate the cognitive deficits observed in AD patients, making them valuable tools for the rapid evaluation of potential therapeutic interventions targeting memory and learning. It has been demonstrated that c-Jun N-terminal kinase (JNK), Akt, and glycogen synthase kinase-3 beta (GSK-3β) play critical roles in the pathophysiology of AD. Phosphorylated JNK (the active form) and dysregulated Akt and GSK-3β have been implicated in the brains of individuals with AD, contributing to the progression of the disease (Mehan et al., 2011; Zhao et al., 2024). It is reported that scopolamine administration induces oxidative stress, which in turn activates JNK and disrupts Akt and GSK-3β signaling (Sandhu et al., 2022; SoukhakLari et al., 2018). Activated JNK and GSK-3β downregulation may contribute to the downregulation of antioxidant proteins, thereby exacerbating synaptic dysfunction, neuroinflammatory responses, and cognitive deficits associated with neurodegeneration. It demonstrates that JNK and GSK-3β could serve as promising therapeutic targets for the management and treatment of AD (Zhou et al., 2015; Yarza et al., 2016; Yang et al., 2016; Li et al., 2023).

Bioactive compounds are strategically utilized to modulate the pathophysiological mechanisms underlying neuronal dysfunction and degeneration, thereby mitigating the morbidity and mortality associated with neurodegenerative disorders (Franco et al., 2023). Plant-derived alkaloids have been shown to possess anti-inflammatory properties by inhibiting various pro-inflammatory protein complexes (Aryal et al., 2022). Ambroxol hydrochloride (2-amino-3,5-dibromo-N-methylbenzylamine hydrochloride), a synthetic derivative of the alkaloid vasicine, a natural alkaloid found in Justicia adhatoda (Rav Ali et al., 2024), is a pharmacological chaperone that has been FDA-approved as an expectorant. It is currently used as an antitussive and anti-asthmatic agent. Beyond its mucolytic effects, ambroxol exhibits antioxidant and anti-inflammatory effects that contribute to its neuroprotective properties. Its potential effects on the central nervous system (CNS) are currently under active investigation (Dhanve et al., 2023; Malerba and Ragnoli, 2008; Patzwaldt et al., 2023). It can cross the blood-brain barrier (BBB) and effectively penetrate the central nervous system (Istaiti et al., 2021). Ambroxol exerts its antioxidant and anti-inflammatory effects through multiple mechanisms (Ahmadi et al., 2024). It mitigates oxidative damage by scavenging free radicals, reducing mitochondrial reactive oxygen species (ROS) production, and enhancing the activity of endogenous antioxidant enzymes (Ullah et al., 2025). Additionally, ambroxol attenuates neuroinflammation and neuronal injury by downregulating pro-inflammatory cytokines, including tumor necrosis factor-alpha (TNF-α) and interleukin-1 beta (IL-1β), inhibiting the nuclear factor kappa B (NF-κB) signaling pathway, and suppressing microglial activation (Faldu and Shah, 2024). Ambroxol mitigates the elevation of phosphorylated c-Jun N-terminal kinase (p-JNK) in the liver and kidney (Bishr et al., 2019). Ambroxol has been reported to inhibit microglial activation and reduce the levels of proinflammatory cytokines in the brain following intracerebral hemorrhage (Jiang et al., 2020).

This study investigates the neuroprotective potential of ambroxol against scopolamine-induced oxidative stress, which triggers JNK activation, disrupts Akt and GSK-3β signaling pathways, and contributes to memory impairments, synaptic dysfunction, neuroinflammation, and neurodegeneration. Our findings demonstrate that ambroxol alleviates scopolamine-induced neuroinflammation, oxidative stress, synaptic dysfunction, and memory impairments in the brains of mice. These neuroprotective effects of ambroxol may be attributed to its inhibition of JNK and GSK-3β alteration, potential interactions with synaptic proteins, and its direct antioxidant and anti-inflammatory properties.

## 2 Materials and methods

### 2.1 Chemicals and antibodies

Scopolamine, ambroxol hydrochloride (CAS 23828-92-4), and 2,7-dichlorodihydrofluorescein diacetate (DCFHDA) were purchased from Sigma-Aldrich (St. Louis, MO, United States), and the primary antibodies were obtained from Santa Cruz Biotechnology (Dallas, TX, United States). An overview of all antibodies utilized in the current study is provided in Table 1.

**TABLE 1:** List of primary antibodies used for western blot and immunofluorescence.

Antibody	Host	Catalog	Application	Dilution	Manufacturer
Nrf-2	Rabbit	12721S	WB/IF	1:1000/1:100	Cell Signaling, United States
Ho-1	Mouse	Sc-136961	WB	1:1000	Santa Cruz Biotechnology, United States
p-JNK	Mouse	Sc-6254	WB/IF	1:1000/1:100	Santa Cruz Biotechnology, United States
p-GSK-3β	Mouse	Sc-373800	WB	1:1000	Santa Cruz Biotechnology, United States
p-Akt	Rabbit	9271S	WB	1:1000	Cell Signaling, United States
GFAP	Mouse	Sc-33673	WB/IF	1:1000/1:100	Santa Cruz Biotechnology, United States
Iba-1	Rabbit	17,198	WB/IF	1:1000/1:100	Cell Signaling, United States
p-NF-κB	Mouse	Sc-136548	WB	1:1000	Santa Cruz Biotechnology, United States
TNF-α	Mouse	Sc-52746	WB	1:1000	Santa Cruz Biotechnology, United States
IL-1	Mouse	Sc-32294	WB	1:1000	Santa Cruz Biotechnology, United States
PSD-95	Mouse	Sc-71933	WB/IF	1:1000/100	Santa Cruz Biotechnology, United States
SNAP-23	Mouse	Sc-374215	WB	1:1000	Santa Cruz Biotechnology, United States
β-Actin	Mouse	Sc-47778	WB	1:1000	Santa Cruz Biotechnology, United States

All the secondary antibodies were diluted in 1x TBST at a concentration of 1:10,000 μL.

### 2.2 Experimental animals

Male wild-type C57BL/6N mice (8 weeks old, with an average body weight of 25–30 g) were obtained from Samtako Bio (Osan, South Korea). Animals were housed and acclimatized for 1 week in the animal care facility under a 12 h light/dark cycle in a temperature-controlled environment (20 ± 2°C; 50 ± 10% humidity) with *ad libitum* access to food and water. All procedures and techniques were conducted following the guidelines established by the Animal Ethics Committee of the Division of Applied Life Sciences, Department of Biology, Gyeongsang National University, South Korea.

### 2.3 Animal grouping, drug administration, and preparation

Four groups, each consisting of eight mice, were randomly selected from the experimental cohort (*n* = 4 for western blot and *n* = 4 for immunohistochemistry).

**FIGURE 1:**
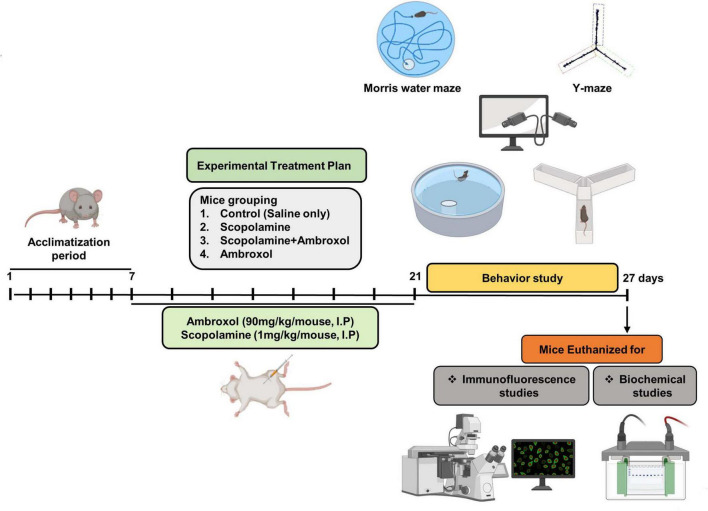
Diagrammatic representation of the treatment regimens, animal groupings, and experimental setup. Following a 7 days acclimatization period, mice were randomly allocated into four experimental groups. The animals received intraperitoneal (i.p.) injections of scopolamine and ambroxol (Amb) for 14 days. Subsequent to behavioral assessments, the animals were euthanized for subsequent biochemical and immunofluorescence analyses.

1. Control (Cont) group: Mice received a 14 days vehicle treatment with normal saline (0.9%, i.p.).
2. Scopolamine (Scop) treated group: Mice were administered scopolamine (1 mg/kg/day, i.p.) dissolved in normal saline for 14 days (Istifo et al., 2024; Muhammad et al., 2019). Seven doses were administered over 2 weeks, on alternate days.
3. Scopolamine + Ambroxol (Scop + Amb) co-treatment group: Mice were treated with scopolamine (1 mg/kg/day) and ambroxol (90 mg/kg/day in 0.9% normal saline) for 14 days. Prepared in different saline solutions and injected separately. Scopolamine was administered in seven doses over 2 weeks, on alternate days. Ambroxol was administered daily for 2 weeks.
4. Ambroxol (Amb) treated group: Mice were treated with ambroxol (90 mg/kg/day in 0.9% normal saline) for 14 days (Su et al., 2004; Patzwaldt et al., 2023). Ambroxol was administered intraperitoneal injection (i.p.) for 14 doses for 2 weeks (Figure 1).

### 2.4 Behavioral study

After a 2 weeks regimen of scopolamine and ambroxol treatment, the mice’s behavioral performance was assessed using the Morris water maze (MWM) and Y-maze paradigms.

#### 2.4.1 Morris water maze test

The Morris Water Maze (MWM) is a widely utilized paradigm for evaluating mice’s memory and learning capabilities. The MWM procedure was conducted with minor modifications, as previously described (Ali et al., 2015). The MWM apparatus consisted of a circular water tank with a diameter of 100 cm and a height of 35 cm. The tank was filled to a depth of 15.5 cm with water maintained at a temperature of 23 ± 1°C. The water was made opaque by applying non-toxic white-colored paint. A transparent escape platform, with a diameter of 4.5 cm and a height of 14.5 cm, was positioned at the center of one quadrant and submerged 1 cm beneath the water’s surface. Each mouse was placed in a designated quadrant and allowed a period of free exploration to locate the submerged platform. The hidden platform was employed for a 4 days consecutive training period for each mouse. During each trial, the escape latency, defined as the time taken to locate the hidden platform, was recorded for each mouse. On day 5, the probe test was conducted to assess memory consolidation. Following platform removal, each mouse was allotted a one-minute interval for free swimming. The probing trial evaluated the number of platform crossings, the duration spent in the target quadrant (the region where the platform was positioned during the hidden platform training), and the time spent in the remaining three quadrants (left, right, and opposite). The duration spent in the target quadrant following the learning phase served as an indicator of memory consolidation. Behavioral data were recorded using visual/video tracking software (SMART, Panlab Harvard Apparatus; Bioscience Company, Holliston, MA, Uited States).

#### 2.4.2 Y-maze test

To evaluate spatial memory, a Y-maze apparatus (length = 50 cm, height = 20 cm, width = 10 cm) equipped with a camera sensor mounted above was employed (Rehman et al., 2017). Each mouse was placed at the center of the apparatus and allowed 8 min for free exploration. The attached sensor digitally recorded the sequence in which each arm was entered. The consecutive entries of each mouse into the three arms in overlapping triplet sets refer to spontaneous alternation. The formula used to determine the percentage of alteration behavior was (number of consecutive triplet sets/total number of arm entries) × 100. Superior cognitive performance is indicated by an increased percentage of spontaneous alternation behavior.

### 2.5 Brain tissue collection and sample preparation

Upon completion of pharmacological treatment and behavioral assessments, all experimental mice (*n* = 32) were euthanized using an intraperitoneal injection of 0.05 mL/100 g body weight of Rompun (Xylazine) and 0.1 mL/100 g body weight of Zoletil (Ketamine). Subsequently, the animals were promptly sacrificed for immunofluorescence and biochemical analyses. The experimental animals (*n* = 16 mice) underwent transcardial perfusion with ice-cold phosphate-buffered saline (PBS, 0.01 M), followed by neutral-buffered paraformaldehyde (NBP, 4%). The brains were then post-fixed in NBP (4%) for 48–72 h before immunofluorescence analysis. After removal of NBP, the post-fixed brains of each experimental mouse were rinsed with 1% PBS (0.01 M) and subsequently immersed in a 20% sucrose solution for 48 h, or until they sank to the bottom of the tube. Before sectioning the cortex and hippocampus (14 μm) using a CM-3050C Cryostat (Leica, Germany), the brains were carefully frozen in O.C.T. compound (A.O., United States), and the sections were then mounted onto microscopic slides. Thawed sections were mounted onto probe-on plus charged slides (Fisher, Rockford, IL, United States) for subsequent analysis. Similarly, for western blotting or biochemical analyses (*n* = 4 mice per group), the brains were swiftly excised, and the cortical and hippocampal tissues were meticulously dissected, snap-frozen on dry ice, and stored at −80°C. As directed by the manufacturer (iNtRON Biotechnology, Inc., Sungnam, South Korea), the tissues were homogenized in a protein extraction solution (PRO-PREP). The samples were centrifuged at 13,000 rpm for 25 min at 4°C, supernatants were carefully collected and stored at −80°C.

### 2.6 Western blot analysis

Western blotting was conducted as previously described, with slight modifications, to quantify the protein concentrations in the cortex and hippocampus (Sarubbo et al., 2018). Protein concentrations were determined using the Bio-Rad Protein Assay Kit (Bio-Rad Laboratories, CA, United States). The protein samples were subsequently subjected to electrophoresis, with equal amounts (15–30 μg) loaded onto 10%/12% Bolt™ Mini Gels (Novex, Life Technologies, Kiryat Shmona, Israel). An anti-β-actin antibody (Santa Cruz Biotechnology, Dallas, TX, United States) was utilized as a loading control to confirm equal loading across the gel. After separation on the gel, the proteins were transferred to polyvinylidene difluoride (PVDF) membranes. To minimize non-specific binding, the membranes were blocked with 5% (w/v) skim milk and incubated with primary antibodies overnight at 4°C. Following incubation with primary antibodies (1:1000) in Tris-buffered saline with Tween (TBST), the membranes were exposed to secondary antibodies for 1–2 h. Subsequently, the membranes were washed three times for 10 min each with 1 × TBST. After incubation with a horseradish peroxidase-conjugated secondary antibody, protein visualization was achieved using the ECL detection reagent (ECL kit; Amersham, Japan) according to the manufacturer’s instructions, and the resulting signals were captured on X-ray films. Following the scanning of the developed X-ray films, histograms were generated by performing densitometric analysis of the bands using ImageJ and GraphPad Prism 6 software. The density values are presented as arbitrary units (A.U.) relative to the untreated control.

### 2.7 Immunofluorescence staining

Immunofluorescence staining was carried out with slight modifications to the previously described protocol (Ali et al., 2015). The 14 μm brain sections on the slides were washed twice with 0.01 M phosphate-buffered saline (PBS) for 10 min each. Subsequently, the slides were incubated for 1 h in a blocking solution composed of 0.3% Triton X-100 in PBS and 2% normal bovine serum, tailored to the specific antibody employed. Following the blocking, primary antibodies (diluted 1:100 in 1% PBS, i.e., 0.01 M) were applied to the slides and incubated overnight at 4°C. The slides were subsequently rinsed with 0.01 M PBS and incubated for 1.5–2 h with secondary antibodies (1:100) conjugated to fluorescein isothiocyanate (FITC, green) or tetramethylrhodamine isothiocyanate (TRITC, red). The slides were then counterstained with DAPI (4′,6-diamidino-2-phenylindole dihydrochloride) for 5–10 min, followed by mounting with glass coverslips using mounting media. The staining patterns were examined and assessed using a confocal laser scanning microscope (Fluoview FV 1000MPE, Olympus, Japan).

### 2.8 Reactive oxygen species (ROS) assay

The reactive oxygen species (ROS) assay was performed with minor modifications to the previously established protocols (Amin et al., 2017; Qian et al., 2008). The assay is primarily based on the oxidation of 2′,7′-dichlorodihydrofluorescein diacetate (DCFH-DA) to 2′,7′-dichlorofluorescein (DCF). Ice-cold Lock’s buffer was used to dilute the cortical and hippocampal brain homogenates at a 1:20 ratio, yielding a final tissue concentration of 2.5 mg per 500 μL. To generate fluorescent DCF from DCFH-DA, the reaction mixture was incubated for 15 min at room temperature, consisting of 1 mL of Lock’s buffer (pH 7.4), 0.2 mL of homogenate, and 10 μL of DCFH-DA (5 mM). The converted fluorescent product, DCF, was measured using a spectrofluorometer, with excitation and emission wavelengths set at 484 nm and 530 nm, respectively. Parallel measurements of blank samples were conducted to account for background fluorescence, which corresponds to the conversion of DCFH-DA in the absence of homogenate. The amount of reactive oxygen species (ROS) present was quantified in picomoles of DCF generated per minute per milligram of protein.

### 2.9 Lipid peroxidation (LPO) assay

As previously described, the lipid peroxidation (LPO) assay is essential for evaluating oxidative stress (Rehman et al., 2017). Following the manufacturer’s protocol, an MDA colorimetric/fluorometric assay kit (BioVision, United States, Cat # K739-100) was utilized to measure free malondialdehyde (MDA) in cortical and hippocampal protein homogenates. MDA was quantified as a marker of lipid peroxidation, serving as an indicator of oxidative lipid degradation. The mouse brains were homogenized in 300 μL of MDA lysis buffer containing 3 μL of butylated hydroxytoluene (BHT), followed by centrifugation at 13,000 rpm for 10 min. To each brain sample, 150 μL of distilled water, 3 μL of BHT, and 1 mL of 2 N perchloric acid were added to precipitate approximately 10 mg of protein. To isolate the precipitated protein, the mixture was vortexed and then centrifuged. The supernatant from each sample was transferred to a 96-well plate, and absorbance was measured at 532 nm using a microplate reader. The total MDA concentration was expressed as nmol of MDA per milligram of protein in each brain homogenate (cortex and hippocampus).

### 2.10 Statistical analysis

Western blot and immunofluorescence images were quantified using ImageJ software for densitometric analysis. The data, presented as the mean ± SEM from eight mice per group, were analyzed using Prism v8 software (GraphPad Software, Inc., San Diego, CA, United States). Group comparisons were performed using a one-way ANOVA, followed by Tukey’s post-hoc test for statistical analysis. A *P*-value less than 0.05 was regarded as statistically significant. The symbol “#” indicates a significant difference compared to the saline-injected group, while the symbol “*” denotes a significant difference relative to the scopolamine-injected group. Significance: #p ≤ 0.05, ##p ≤ 0.01, and ###p ≤ 0.001; *p ≤ 0.05, **p ≤ 0.01, and ***p ≤ 0.001.

## 3 Results

### 3.1 Ambroxol rescues scopolamine-induced oxidative stress and enhances Nrf-2/HO-1 expression in the mouse brain

**FIGURE 2:**
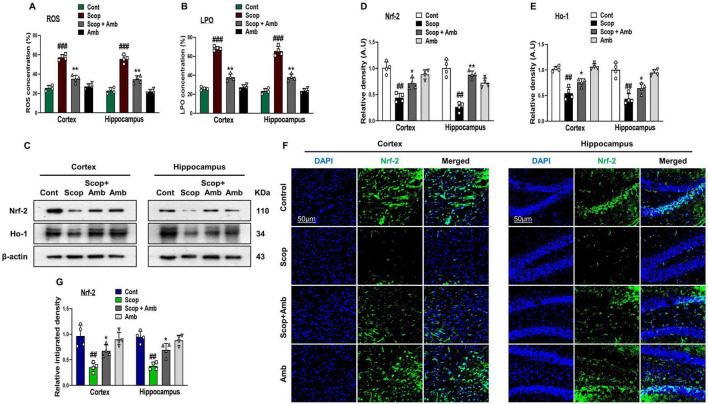
Ambroxol reduced reactive oxygen species (ROS) and lipid peroxidation (LPO) levels and concurrently upregulated the expression of nuclear factor erythroid 2-related factor 2 (Nrf-2) and heme oxygenase-1 (HO-1) in the brains of scopolamine-treated mice. **(A,B)** Representative histograms of ROS and LPO assays in the mice brain cortex and hippocampus. **(C)** Images of western blot analysis illustrating the protein expression levels of Nrf-2 and HO-1 in the cortex and hippocampus. **(D,E)** Histograms of Nrf-2 and HO-1 protein expression. **(F)** Immunofluorescence images depicting the immunoreactivity of Nrf-2 protein in the cortex and hippocampus. **(G)** Corresponding bar graphs of Nrf-2 immunofluorescence. β-actin was used as the loading control. Band intensities were cropped and quantified using ImageJ software, and the variations are illustrated in the corresponding histogram; Magnification 10×. Scale bar 50 μm. All data are presented as mean ± S.E.M, with corresponding bar graphs. An asterisk (*) denotes a significant difference from the normal saline-treated group; hash (#) indicates a significant difference from the scopolamine-treated group. Significance: *P ≤ 0.05, **P ≤ 0.01, ***P ≤ 0.001; #*P* ≤ 0.05, ##*P* ≤ 0.01; ###*P* ≤ 0.001.

Scopolamine has been connected to neurodegeneration caused by oxidative stress (Gul et al., 2023; Muhammad et al., 2019; Skalicka-Wozniak et al., 2018; Ponne et al., 2020). While ambroxol exhibits antioxidant properties, mitigating oxidative stress-induced pathologies (Štětinová et al., 2004; Jiang et al., 2013; Cavalu et al., 2022). To assess the neuroprotective potential of ambroxol against scopolamine-induced oxidative brain damage in mice, we quantified reactive oxygen species (ROS) levels and lipid peroxidation (LPO) in hippocampal and cortical tissue homogenates from the experimental groups. Our findings revealed that scopolamine administration markedly increased LPO and ROS levels in cortical and hippocampal homogenates of scopolamine-treated mouse brains compared to saline-treated. Notably, ambroxol treatment significantly attenuated ROS and LPO levels in cortical and hippocampal homogenates of ambroxol + scopolamine co-treated mouse brains (Figures 2A, B). Furthermore, the antioxidant efficacy of ambroxol against scopolamine-induced oxidative stress in the mice brain was confirmed by assessing the expression levels of key antioxidant proteins, including nuclear factor erythroid 2-related factor 2 (Nrf-2) and heme oxygenase-1 (HO-1), via western blot analysis. The immunoblot analysis revealed a reduction in the expression levels of Nrf-2 and HO-1 in the cerebral tissues of scopolamine-administered mice. In contrast, treatment with ambroxol notably enhanced the expression of these antioxidant proteins in the brains of mice co-treated with ambroxol + scopolamine (Figures 2C–E). Furthermore, we performed an immunofluorescence analysis to validate the immunoblotting findings for Nrf-2, offering spatial insights into its subcellular localization within the nucleus. Immunofluorescence analysis demonstrated a reduction in Nrf-2 reactivity in both the cortex and hippocampus of scopolamine-treated mice, whereas a significant upregulation of Nrf-2 expression was observed in the ambroxol + scopolamine co-treated group (Figures 2F, G). Collectively, these findings suggest that ambroxol mitigates scopolamine-induced oxidative stress in the mouse brain, likely via its antioxidant properties.

### 3.2 Ambroxol exerts neuroprotective effects against scopolamine-induced neurotoxicity by modulating the JNK, Akt, and GSK-3β signaling in the mouse brain

**FIGURE 3:**
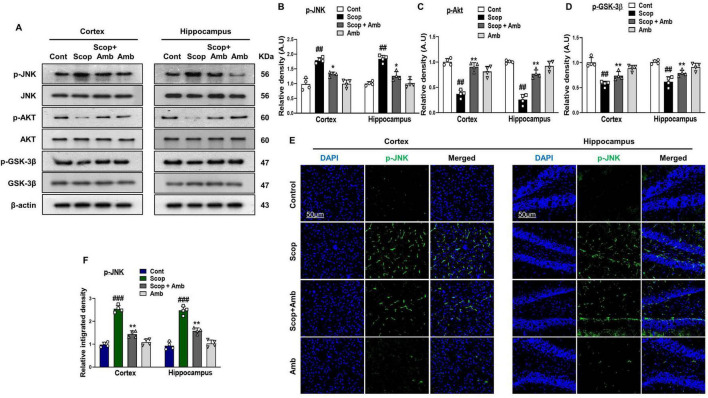
Ambroxol modulates the expression of p-JNK, p-Akt, and p-GSK-3β in the mouse brain, thereby alleviating scopolamine-induced stress. **(A)** Images of western blot analysis illustrating the protein expression levels of p-JNK, p-Akt, and p-GSK-3β in the cortex and hippocampus. **(B–D)** Histograms of p-JNK, p-Akt, and p-GSK-3β protein expression. **(E)** Immunofluorescence images depicting the immunoreactivity of p-JNK protein in the cortex and hippocampus. **(F)** Corresponding bar graphs of p-JNK immunofluorescence. β-actin was used as the loading control. Band intensities were cropped and quantified using ImageJ software, and the variations are illustrated in the corresponding histogram; Magnification 10x. Scale bar 50 μm. All data are presented as mean ± S.E.M, with corresponding bar graphs. An asterisk (*) denotes a significant difference from the normal saline-treated group; hash (#) indicates a significant difference from the scopolamine-treated group. Significance: *P ≤ 0.05, **P ≤ 0.01, ***P ≤ 0.001; #*P* ≤ 0.05, ##*P* ≤ 0.01; ###*P* ≤ 0.001.

An increasing amount of evidence revealed the involvement of c-Jun N-terminal kinase (JNK), also known as stress-activated protein kinases, in a range of pathophysiological processes underlying AD (Mehan et al., 2011; Zhou et al., 2015). The expression level of the phosphorylated JNK (p-JNK) protein was assessed via western blot to evaluate the effect of scopolamine-induced oxidative stress on the activation of stress kinases. The protein levels of JNK were significantly increased in the scopolamine-treated group, whereas ambroxol effectively attenuated the elevated production of phosphorylated JNK (p-JNK) (Figures 3A–D). Similarly, western blot analysis was employed to investigate whether ambroxol could restore the dysregulation of GSK-3β induced by scopolamine treatment. Glycogen synthase kinase-3β (GSK-3β) is a ubiquitously expressed, constitutively active serine/threonine kinase that plays a critical role in the regulation of numerous fundamental cellular pathways. It contributes to the pathophysiology of AD through multiple distinct mechanisms. Dysregulation of this kinase has been implicated in both *in vitro* and *in vivo* models of AD. It has been reported that scopolamine downregulates the expression of GSK-3β (Lauretti et al., 2020; Llorens-Marítin et al., 2014; SoukhakLari et al., 2018). GSK-3β is a key target of Akt, which induces its phosphorylation at the serine 9 position, leading to its inactivation. Previously, studies have reported that scopolamine-induced Akt deactivation and GSK-3β alteration (SoukhakLari et al., 2018; Muhammad et al., 2019). Our findings demonstrated a significant reduction in the protein levels of Akt and GSK-3β alteration in both the cortical and hippocampal regions of mice treated with scopolamine, compared to those treated with the control vehicle. However, ambroxol treatment significantly elevated the levels of Akt and GSK-3β in both the cortex and hippocampus (Figure 3A). Moreover, immunofluorescence analysis demonstrated a significant increase in p-JNK immunoreactivity in the cortex and hippocampus of scopolamine-treated mice, compared to the control saline-treated group. Interestingly, these elevated fluorescence levels were reduced in the brains of scopolamine + ambroxol co-treated mice, indicating that ambroxol effectively prevented scopolamine-induced JNK activation (Figures 3E, F).

### 3.3 Ambroxol mitigates scopolamine-induced neuroinflammation and attenuates the activation of glial cells

**FIGURE 4:**
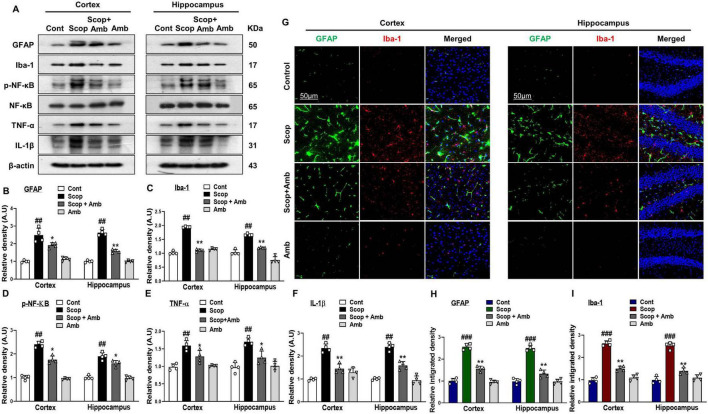
Ambroxol inhibited scopolamine-induced glial cell activation and suppressed the upregulation of inflammatory proteins in the mice brain. **(A)** Images of western blot analysis illustrating the protein expression levels of GFAP, Iba-1, p-NF-κB (p-p65), TNF-α, and IL-1β in the cortex and hippocampus. (**B–F)** Histograms of GFAP, Iba-1, p-NF-κB (p-p65), TNF-α, and IL-1β protein expression. **(G)** Immunofluorescence images depicting the immunoreactivity of GFAP and Iba-1 protein in the cortex and hippocampus. **(H,I)** Corresponding bar graphs of GFAP and Iba-1 immunofluorescence. β-actin was used as the loading control. Band intensities were cropped and quantified using ImageJ software, and the variations are illustrated in the corresponding histogram; Magnification 10x. Scale bar 50 μm. All data are presented as mean ± S.E.M, with corresponding bar graphs. An asterisk (*) denotes a significant difference from the normal saline-treated group; hash (#) indicates a significant difference from the scopolamine-treated group. Significance: *P ≤ 0.05, **P ≤ 0.01, ***P ≤ 0.001; #*P* ≤ 0.05, ##*P* ≤ 0.01; ###*P* ≤ 0.001.

Astrocytes and microglia play a pivotal role in both neuroinflammation and inflammatory neurodegeneration, serving as primary sources of various pro-inflammatory cytokines (Combs, 2009). Iba-1 and GFAP are well-established markers used to identify microglia and inflammatory astrocytes, respectively (Ahmad et al., 2024; Ali et al., 2024). We investigated the protective effect of ambroxol against ionized calcium-binding molecule 1 (Iba-1) and glial fibrillary acidic protein (GFAP) in the cortex and hippocampus, which serve as key markers of reactive astrocytes and microglia. Western blot analysis revealed increased expression levels of Iba-1 and GFAP in the brains of scopolamine-treated mice. However, co-treatment with ambroxol effectively reduced the levels of these proteins (Figure 4A). Additionally, immunofluorescence staining corroborated the western blot results for GFAP and Iba-1, revealing an increased intensity of GFAP and Iba-1-positive cells in the brains of scopolamine-treated mice. Importantly, ambroxol treatment in combination with scopolamine significantly reduced the number of activated GFAP and Iba-1 positive cells in the cortical and hippocampal regions, compared to scopolamine treatment alone (Figures 4G, I). Previous studies have implicated an imbalance in the nuclear factor kappa-light-chain-enhancer of activated B cells (NF-κB) in the pathogenesis of several neurodegenerative diseases, including Alzheimer’s, Parkinson’s, and Huntington’s diseases. Furthermore, the expression of NF-κB has been shown to increase with aging (Sun et al., 2022; Hu et al., 2022; Calabrese et al., 2011; Ahmad et al., 2024). Consistent with previous reports, our results demonstrated that ambroxol co-treatment significantly attenuated the elevated expression of p-NF-κB (p-p65). Overexpression of NF-κB may trigger the activation of several pro-inflammatory markers, including tumor necrosis factor-alpha (TNF-α) and interleukin-1β (IL-1β), which are implicated in the progression of neurodegeneration (Ali et al., 2023). The levels of activated inflammatory markers, including TNF-α and IL-1β, were assessed in cortical and hippocampal samples from scopolamine-treated mice using western blot analysis. Our results demonstrated that ambroxol significantly mitigated scopolamine-induced neuroinflammation by reducing the expression levels of TNF-α and IL-1β (Figures 4A–F).

### 3.4 Ambroxol ameliorated scopolamine-induced synaptic dysfunction and improved memory performance

**FIGURE 5:**
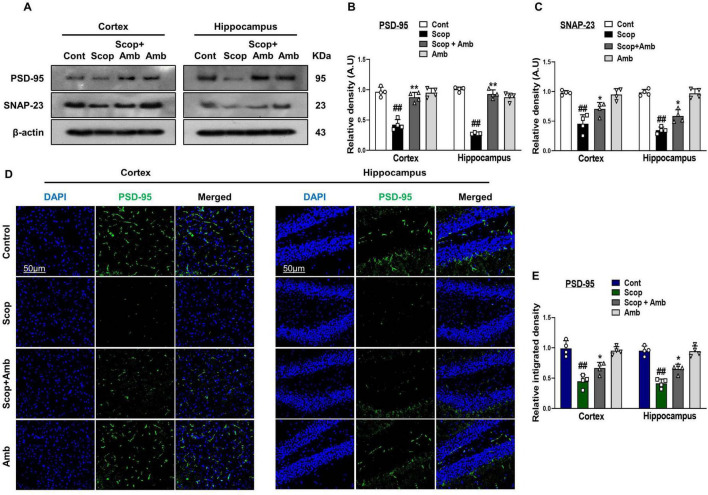
Effect of Ambroxol on scopolamine-induced synaptic dysfunction and memory impairment in mice brain. **(A)** Images of western blot analysis illustrating the protein expression levels of PSD-95 and SNAP-23 in the cortex and hippocampus. **(B,C)** Histograms of PSD-95 and SNAP-23 protein expression. **(D)** Immunofluorescence images depicting the immunoreactivity of PSD-95 protein in the cortex and hippocampus. **(E)** Corresponding bar graphs of PSD-95 immunofluorescence. β-actin was used as the loading control. Band intensities were cropped and quantified using ImageJ software, and the variations are illustrated in the corresponding histogram; Magnification 10×. Scale bar 50 μm. All data are presented as mean ± S.E.M, with corresponding bar graphs. An asterisk (*) denotes a significant difference from the normal saline-treated group; hash (#) indicates a significant difference from the scopolamine-treated group. Significance: *P ≤ 0.05, **P ≤ 0.01, ***P ≤ 0.001; #*P* ≤ 0.05, ##*P* ≤ 0.01; ###*P* ≤ 0.001.

Neuronal and synaptic dysfunction are key consequences of AD neuropathology and are strongly linked to cognitive decline and memory impairment (Oddo et al., 2003). To assess the protective effects of ambroxol against scopolamine-induced synaptic loss, we examined the expression of synaptic proteins in the cortical and hippocampal tissues of scopolamine-treated mice using western blot analysis and confocal microscopy. Our findings demonstrated that scopolamine significantly reduced the expression levels of synaptic proteins, such as postsynaptic density protein (PSD-95) and synaptosomal-associated protein 23 (SNAP-23), compared to the control group. However, ambroxol treatment significantly upregulated the expression of these proteins, thereby markedly enhancing synaptic protein levels in the cortical and hippocampal tissues of scopolamine-treated mice (Figures 5A–C). Furthermore, we employed an immunofluorescence assay for postsynaptic density protein (PSD-95) to corroborate the western blot data. Immunofluorescence results showed that scopolamine treatment significantly decreased postsynaptic density protein (PSD-95) levels compared to the control group. However, ambroxol co-treatment notably restored the levels of PSD-95 (Figures 5D, E).

### 3.5 Ambroxol attenuates scopolamine-induced behavioral and cognitive deficits in mice

**FIGURE 6:**
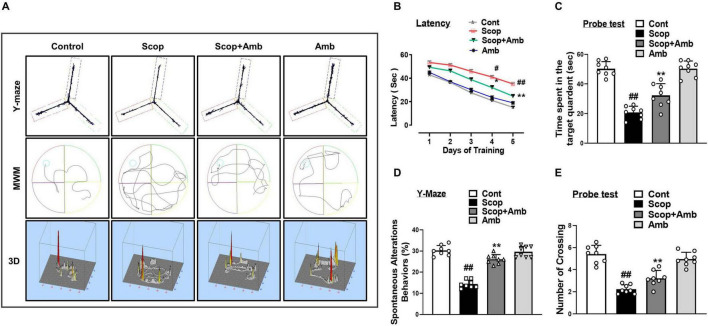
Ambroxol enhances learning memory and improves spontaneous alteration behavior in mice with scopolamine-induced memory impairment. **(A)** Trajectories of Y-maze and Morris water maze analysis. **(B)** Mean escape latency to reach the hidden platform during the training sessions. **(C)** Time spent in the platform quadrant, where the hidden platform was located, during the trial session. **(D)** The percentage of spontaneous alteration behavior during the Y-maze analysis. **(E)** The average number of target crossings at the hidden platform during the Morris water maze (MWM) test probe trial. For the behavioral study, each experimental group consisted of eight mice (*n* = 8). All data are presented as mean ± S.E.M, with corresponding bar graphs. An asterisk (*) denotes a significant difference from the normal saline-treated group; hash (#) indicates a significant difference from the scopolamine-treated group. Significance: *P ≤ 0.05, **P ≤ 0.01, ***P ≤ 0.001; #*P* ≤ 0.05, ##*P* ≤ 0.01; ###*P* ≤ 0.001.

The neuroprotective effects of ambroxol on spatial memory were evaluated in mice exhibiting cognitive and behavioral deficits induced by scopolamine. These effects were assessed using the Morris Water Maze (MWM) and Y-maze paradigms. In the MWM, mice from each experimental group were trained for 5 days to evaluate their learning abilities by practicing with a hidden platform. For the training period, the mean latency to locate the hidden platform steadily dropped. In contrast to the control group, the scopolamine-treated group showed a greater delay in locating the platform, suggesting impaired spatial learning and memory. Our findings demonstrated that administering ambroxol considerably reduced the elevated latency to find the platform during the training days brought on by scopolamine treatment. On day 6, we removed the platform and conducted a probe test to evaluate memory formation. Mice treated with scopolamine exhibited considerably fewer crossings and spent less time in the target quadrant than control mice. However, in the ambroxol co-treated group, both the number of platform crossings and the time spent in the target quadrant were significantly increased compared to the scopolamine-only treated group (Figures 6A–C). To further examine the cognitive abilities of mice, we assessed exploratory behavior and spatial working memory, a type of short-term memory, by determining the percentage (%) of spontaneous alternation behavior using the Y-maze test. An increase in the percentage of spontaneous alternation behavior was taken as an indication of enhanced memory function. Analysis of the spontaneous alternation rate from the center of the Y-maze is based on the percentage of spontaneous alternation behaviors, evaluated in the form of the total number of arm entries, a measure of exploratory activity, and successive triplets. Our results indicated that scopolamine-treated mice had a lower percentage of alternation than control mice, thus exhibiting reduced working memory. In contrast, the ambroxol + scopolamine-treated group significantly increased spontaneous alternation behavior (%) compared to the scopolamine-treated group, indicating that ambroxol mitigated memory deficits in the scopolamine-administered mice model. These findings provide evidence that ambroxol treatment not only increased exploratory behavior but also alleviated scopolamine-induced cognitive dysfunction (Figures 6A, D, E).

## 4 Discussion

Alzheimer’s Disease (AD) stands as the most prevalent and predominant cause of dementia (Sequeira and Godad, 2024). Although the exact cause of AD is still unknown, oxidative stress and mitochondrial dysfunction are considered key factors in the disease’s progression (Tichon et al., 2023). It is characterized by neuronal degeneration, synaptic disruption, and behavioral anomalies, which subsequently lead to memory deficits and a progressive decline in cognitive functions (Rummel et al., 2022). The antimuscarinic agent scopolamine has been employed to antagonize muscarinic receptors, thereby inducing AD-like dementia in various animal models, which is known to induce oxidative stress, a key pathological feature in the development of AD.(Mostafa et al., 2021). Increased oxidative stress triggers a cascade of events that leads to synaptic dysfunction, neuroinflammation, and neurodegeneration, collectively contributing to the progression of neurodegenerative diseases (Tönnies and Trushina, 2017).

We investigated the neuroprotective effects of ambroxol against scopolamine-induced oxidative stress mouse model of AD. Nrf-2, a key regulator of endogenous antioxidant gene expression, acts as a stress-responsive transcription factor that mitigates reactive oxygen species (ROS)-induced oxidative stress across various pathological conditions.

Under normal physiological conditions, Nrf-2 is bound to its cytoplasmic inhibitor Keap1, facilitating its polyubiquitination and subsequent proteasomal degradation. However, under normal oxidative stress (moderate, acute), the Keap1-dependent ubiquitin ligase activity is inhibited, allowing Nrf-2 to escape degradation, translocate to the nucleus, which subsequently triggers the activation of other redox-regulated enzymes, such as heme oxygenase-1 (HO-1), to counteract the damaging effects of oxidative stress (Chen et al., 2012; Jana et al., 2018; Kim et al., 2020). However, previous studies have demonstrated that scopolamine-induced oxidative stress is more severe and sustained, which can impair the Nrf-2 signaling pathway and suppress the expression of Nrf-2 and HO-1, rather than activating it, thereby impairing the cellular defense mechanisms against oxidative damage and being involved in the pathogenesis of AD (Li et al., 2024; Ju et al., 2021; Yoo et al., 2024; Venkatesan et al., 2016; Subedi et al., 2019; Balakrishnan et al., 2023). According to reactive oxygen species (ROS) and lipid peroxidation (LPO) assays, ambroxol treatment significantly reduced ROS and LPO levels in the brains of mice subjected to scopolamine-induced oxidative stress. Furthermore, our findings demonstrate that ambroxol administration effectively reversed the scopolamine-induced effects, significantly increasing the expression levels of Nrf-2 and HO-1 proteins. Ambroxol alleviates scopolamine-induced oxidative stress and enhances the cellular antioxidant defense mechanism.

The c-Jun N-terminal kinase (JNK), a member of the mitogen-activated protein kinase (MAPK) family, is a highly conserved signal transduction pathway that can be activated by a diverse array of environmental stimuli. It plays a crucial role in regulating a variety of physiological processes (Cargnello and Roux, 2011; Gehi et al., 2022). Previous reports demonstrated that oxidative stress activates the JNK signaling pathway, resulting in prolonged phosphorylation of JNK, which disrupts cellular homeostasis (Mehan et al., 2011; Zhu et al., 2001; Quiroz-Baez et al., 2009). Glycogen synthase kinase-3β (GSK-3β) is a widely expressed and constitutively active serine/threonine kinase that plays a pivotal role in regulating numerous essential cellular pathways. It contributes to the pathogenesis of AD through multiple distinct mechanisms (Lauretti et al., 2020). It has been reported that oxidative stress plays a central role in disrupting GSK-3β, which in turn contributes to the key pathological features of AD (Dhapola et al., 2024; Lauretti et al., 2020). Accordingly, our findings indicated that scopolamine increased the expression levels of p-JNK and disrupted GSK-3β; however, ambroxol therapy significantly restores these proteins’ expression. Therefore, by inhibiting the JNK and restoring GSK-3β pathways, our results propose a novel and distinct neuroprotective mechanism of ambroxol in counteracting scopolamine-induced oxidative stress.

Phosphorylated JNK (p-JNK) and GSK-3β, in addition to their pivotal roles in regulating physiological processes and key cellular pathways, serve as crucial mediators of microglial activation and neuroinflammation (Wang et al., 2010; Biswas, 2023). Chronic neuroinflammation is a potential risk factor for various age-related diseases (Sanada et al., 2018). Glial cells, including microglia and astrocytes, constitute the frontline defense of the central nervous system and play a central role in amplifying the neuroinflammatory response in neurodegenerative disorders (Singh, 2022). Ionized calcium-binding adaptor molecule 1 (Iba-1) and glial fibrillary acidic protein (GFAP) are specific markers of microglia and astrocyte activation (Norden et al., 2016). Previously, it has been reported that scopolamine administration induces activation of Iba-1 and GFAP (Muhammad et al., 2019). According to earlier research, activated glial cells overexpress the transcription factor NF-κB, leading to increased production of various proinflammatory mediators, such as TNF-α and IL-1β, which in turn contributes to neuroinflammation (Ahmad et al., 2024; Maqbool et al., 2013). Here, we found that scopolamine treatment induces the expression level of GFAP and Iba-1 in the cortex and hippocampus. In contrast, co-treatment of scopolamine and ambroxol markedly reduced the protein expression and immunofluorescence reactivity of GFAP and Iba-1 in the cortex and hippocampus. We also found that scopolamine enhances p-NF-κB (p-p65) and proinflammatory mediators such as TNF-α and IL-1β expression compared to saline-treated mice, which was significantly reversed by scopolamine + ambroxol treatment. These findings demonstrate that ambroxol treatment effectively inhibits the activation of glial cells and neuroinflammatory mediators, thereby preventing scopolamine-induced neuroinflammation.

Previously, it has been reported that oxidative stress, JNK, and GSK-3β signaling are involved in learning and memory deficits (Ullah et al., 2020; Takashima, 2006). Control of synaptic plasticity by synaptic proteins is essential for brain function (Lu et al., 2021). Scopolamine-induced synaptic and memory impairment by downregulating memory-associated synaptic protein levels in mice brains (Li et al., 2021; Zhang et al., 2022). Our results showed that scopolamine treatment reduced the expression levels of synaptic proteins, whereas cotreatment with ambroxol recovered the reduced synaptic protein levels. Furthermore, we found that scopolamine dramatically decreased memory function as measured by the Y-Maze and MWM tests. In the MWM test, ambroxol administration decreased the escape latency and increased the number of platform crossings and time spent by the mice in the target quadrant. An increase in the percentage (%) of spontaneous alteration behavior was detected in the Y-maze test. These results show that ambroxol treatment reduces scopolamine-induced synaptic protein loss and spatial learning/cognitive impairment, improving behavior and memory. We propose that ambroxol therapy is effective against scopolamine-induced cognitive and memory impairment.

## Conclusion

**FIGURE 7:**
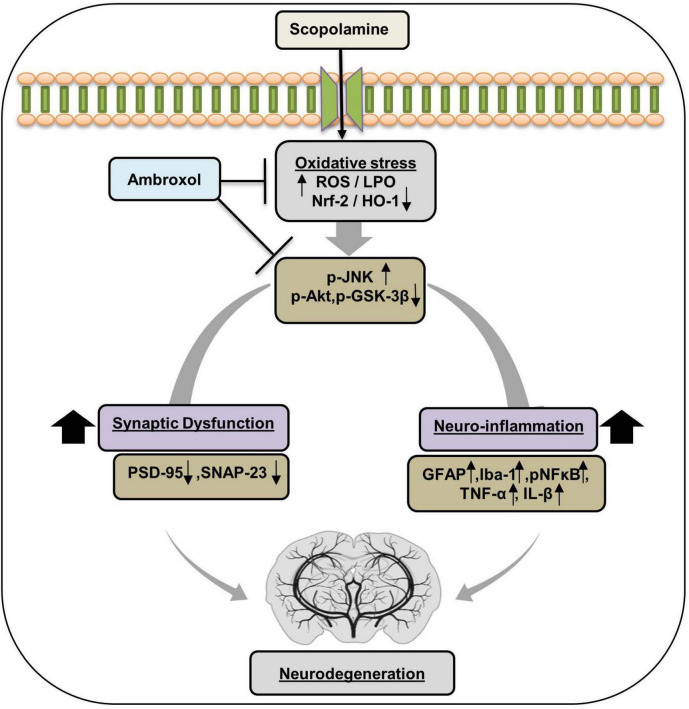
Diagrammatic representation of Ambroxol’s suggested neuroprotective mechanism against oxidative stress, neuroinflammation, synaptic dysfunction, and memory impairment against scopolamine.

Ambroxol alleviates scopolamine-induced oxidative stress, modulates neuroinflammatory pathways, and mitigates neurodegenerative changes. It reduces lipid peroxidation (LPO) and reactive oxygen species (ROS) production while upregulating the expression of key antioxidant defense proteins. Ambroxol inhibits c-Jun N-terminal kinase (JNK) activation and restores dysregulated Akt and GSK-3β signaling pathways. Additionally, it attenuates activated gliosis and downregulates inflammatory mediators. Furthermore, ambroxol promotes synaptic integrity and facilitates cognitive recovery (Figure 7).

## Data Availability

The original contributions presented in this study are included in this article/Supplementary material, further inquiries can be directed to the corresponding author.
